# Bioactive Compounds and Antioxidant Activity in Seeds of Bred Lines of Common Bean Developed from Interspecific Crosses

**DOI:** 10.3390/foods12152849

**Published:** 2023-07-27

**Authors:** Juan Carlos Suárez, Paola Andrea Polanía-Hincapié, Sebastian Saldarriaga, Vivian Yorlady Ramón-Triana, Milan O. Urban, Stephen E. Beebe, Idupulapati M. Rao

**Affiliations:** 1 Programa de Ingeniería Agroecológica, Facultad de Ingeniería, Universidad de la Amazonia, Florencia 180001, Colombia; 2 Centro de Investigaciones Amazónicas CIMAZ Macagual César Augusto Estrada González, Grupo de Investigaciones Agroecosistemas y Conservación en Bosques Amazónicos—GAIA, Florencia 180001, Colombia; v.ramon@udla.edu.co; 3 Programa de Maestría en Sistemas Sostenibles de Producción, Facultad de Ingeniería, Universidad de la Amazonia, Florencia 180002, Colombia; pa.polania@udla.edu.co; 4 Centro de Investigaciones Amazónicas CIMAZ Macagual César Augusto Estrada González, Grupo de Investigación de Productos Naturales Amazónicos, Florencia 180001, Colombia; s.saldarriaga@udla.edu.co; 5 International Center for Tropical Agriculture (CIAT), Km 17 Recta Cali-Palmira, Cali 763537, Colombia; m.urban@cgiar.org (M.O.U.); s.beebe@cgiar.org (S.E.B.); i.rao@cgiar.org (I.M.R.)

**Keywords:** phenolic content, flavonoids, anthocyanin, antioxidant activity, DPPH, FRAP, common bean

## Abstract

Knowledge is limited about the level of bioactive compounds and antioxidant activity of seeds from bred lines of common beans developed from interspecific crosses using four different *Phaseolus* species (*P. vulgaris* L., *P. coccineus* L., *P. acutifolius* A. Gray. Gray., and *P. dumosus*). In this study, differences in the nutritional quality of seeds among 112 bean genotypes were evaluated by measuring the levels of phenolic compounds, pigments, antioxidant activity, and sugars. The bean genotypes were grown under high temperatures and acid soil conditions in the Amazon region of Colombia. Five typology groups of bean genotypes were identified based on the level of bioactive compounds and their functional capacity: (1) highly bioactive and functional (HBF); (2) moderately bioactive and functional (MBF); (3) moderate antioxidant content with pigment influence (MACP); (4) moderately antinutritional with limited antioxidant potential (MALAP); and (5) antinutritional, low bioactive, and functional (ALBF). We developed a nutritional quality index (NQI) with values ranging from 0 to 1 based on the nutritional and anti-nutritional balance of each genotype and the higher values of the NQI of a genotype indicating greater nutritional quality. We found three interspecific bred lines (SER 212, SER 213, and RRA 81), with NQI values higher than 0.8. These three lines belong to the typology group of HBF. The superior nutritional quality of these three interspecific bred lines is attributed to a greater level of bioactive compounds and antioxidant capacity. These three bred lines may serve as useful parents to develop nutritionally superior and stress-resilient beans from bean breeding programs. Further research is needed to explore the role of testa color in improving the nutritional quality of seeds of common bean genotypes grown under different climatic conditions.

## 1. Introduction

Grain legumes contribute significantly to world food security [1] and these are the second most consumed food crops after cereals [2]. Legume seeds have high potential for nutritional quality improvement. The genus *Phaseolus* belongs to the family Fabaceae, which contains more than 400 species. As cultivated forms, *Phaseolus vulgaris* L., *Phaseolus coccineus* L., *Phaseolus lunatus* L., *Phaseolus acutifolius* A. Gray., and *Phaseolus dumosus* Macfady are important [3] for crop improvement. All five species have their origin, domestication, and diversification in the Americas. Only the first three species have a worldwide distribution [4,5].

The common bean (*P. vulgaris*) constitutes one of the basic products of the human diet [6] and its cultivation is recognized as one of the most important agricultural activities in different regions of Colombia, representing a major economic and employment source in rural areas [7,8]. The runner bean (*P. coccineus*) ranks second in terms of bean consumption and like the year-long bean (*P. dumosus*), these two bean types stand out for some of their desirable nutritional and agronomic characteristics including their adaptation to biotic constraints prevalent in humid environments in lowland tropics [9,10,11]. Likewise, the tepary bean (*P. acutifolius*) is an edible bean adapted to drought and high temperatures and is also resistant to some pests and diseases, while its nutritional quality is not high [1,12].

In addition to providing food security, beans contribute to greater nutritional security through bioactive compounds [13], which include metabolites such as polyphenols (phenols, flavonoids, anthocyanins) [14,15] and sugars [16], as well as from their antioxidant activity and anti-nutritional agents, such as trypsin, tannins, and lectins [3,17]. These bioactive compounds are not only essential for the nutritional quality of the grain but also play an important role in human health [9]. These compounds are associated with the ability to restore the oxidative balance in the organism and are the main precursors of amino acids and peptides, thereby becoming essential components of food systems by representing a means of antioxidant defense in tissues [18]. Different authors highlighted the desirable nutritional traits of beans including antiglycemic, antiobesity, anti-inflammatory, antimutagenic, and anti-carcenogenic properties [13,19,20]. Consumption of beans also reduces the risk of cardiovascular diseases; neurodegenerative diseases such as Alzheimer’s and Parkinson’s; stress; anxiety; depression; and digestive tract diseases [21,22].

Previous research on the physiological and agronomic evaluation of a wide range of bean genotypes grown under high temperatures and acid soil stress conditions in the Colombian Amazon resulted in the identification of a few promising interspecific and intraspecific bred lines of common bean [23,24,25,26]. These include four bred lines (BFS 10, GGR 147, SMG 12, SMG 21) that showed grain yields exceeding 1800 kg ha^−1^ compared to genotypes such as VAX 1, EMP 509, and RADICAL with yields of less than 500 kg ha^−1^ [23,24]. There is limited knowledge of the nutritional quality characteristics of bean genotypes adapted to high temperatures and acid soil stress conditions and this nutritional evaluation in seeds is needed to support the ongoing breeding efforts to improve the yield and nutritional quality of common beans under stressful environments [13,23,25,26]. The importance of this study for the western region of the Colombian Amazon lies in the identification of bean genotypes that combine greater agronomic performance with superior nutritional quality in the grain to help the rural infant population in the region suffering from malnutrition and anemia that reduce the development and growth of children [25,26,27]. This study aimed to evaluate the concentration of bioactive compounds such as polyphenols and the content of total and reducing sugars in bred lines resulting from interspecific crosses. We tested the hypothesis that improved adaptation to high temperature and acid soil stress conditions can also improve the nutritional quality of seeds in some bean genotypes.

## 2. Materials and Methods

### 2.1. Plant Material

Mature seeds from a set of 112 genotypes resulting from different crosses using *P. vulgaris*, *P. coccineus*, *P. dumosus*, and *P. acutifolius* species were tested for bioactive compounds and antioxidant activity. Seeds of the different bean lines evaluated were collected from three field experiments conducted by Suárez et al. [24,28,29]. The bean lines evaluated showed adaptation to acid soils and high temperatures.

The seeds were harvested from plants (*n* = 180) from field trials conducted at the Macagual Research Center of the Universidad de la Amazonia, Colombia (1°37′ N and 75°36′ W), which is located 24 km from the municipality of Florencia. The climatic conditions present in the study area (tropical rainforest ecosystem) correspond to an average annual rainfall of 3800 mm, an average temperature of 25.5 °C, and a relative humidity of 84% with 1700 h of sunshine per year. Among the genotypes evaluated include 46 *Phaseolus vulgaris* lines (6 Andean and 40 Mesoamerican); 5 lines derived from the cross of *P. vulgaris* × *P. acutifolius*; 1 line derived from the cross of *P. vulgaris* × *P. acutifolius* × *P. parvifolius*; 19 lines from the cross of *P. vulgaris* × *P. coccineus*; 13 lines from the cross of *P. vulgaris* × *P. acutifolius* × *P. coccineus*; 5 lines from the cross of *P. vulgaris* × *P. acutifolius* × *P. coccineus* × *P. dumosus*; and 1 line derived from the cross of *P. vulgaris* × *P. dumosus*. Similarly, 22 accessions of *P. acutifolius* were evaluated: 16 accessions of *P. acutifolius* var. acutifolius (Cultivated), 4 accessions of *P. acutifolius* var. acutifolius (Wild), and 2 accessions of *P. acutifolius* var. tenuifolius (Wild) (Supplementary Table S1). These lines constitute breeding materials with good agronomic potential, due to their resistance to biotic and abiotic stress factors, as well as to improved grain and nutritional quality.

The list of genotypes evaluated in this study includes ALB lines (small red kidney, black kidney) and BFS lines (small red), adapted to drought and to soils with low fertility and high acidity; the DAA (fuchsia-red) Andean bean line adapted to drought; DAB (speckled-red, red-pink) drought-adapted Andean lines selected for seed color and size; the INB line, an interspecific progeny between the tepary bean and common bean, with some level of resistance to common bacterial blight; RRA lines with resistance to root rot caused by *Pythium* and *Sclerotium*; SAB lines of cream and red mottled seeds of medium size with heat and drought resistance; the SCR (red) line and the commercial variety DOR 390 (black) line with recessive genes for common mosaic virus; SEF (red) heat and drought adapted, SER (red), SEN (black), SXB (cream), and SIN lines with adaptation to drought; SMC (brown) with high mineral (Fe and Zn) content and drought adaptation, the SMG (pink) line with high mineral content, and the long seed with drought adaptation; SMN (black) and SMR (red) lines with high mineral content and adaptation to drought; the VAP line derived from the cross of *P. vulgaris* × *P. acutifolius* × *P. parvifolius* with heat tolerance; and the VAX-1 line (mottled-cream), sensitive to Al toxicity. Commercial and old varieties such as ICA QUIMBAYA (red) and CALIMA (mottled red), which are resistant to aluminum, and the commercial line AMADEUS (bright light red), adapted to drought, were used as checks.

### 2.2. Preparation of Methanolic Extracts

To a 2.0 g sample of bean seed meal, previously dehydrated (dry weight, dw) and ground to a particle size of 0.45 mm, 10.0 mL of 99.9% (*v*/*v*) analytical grade methanol was added and left for a period of 8 days with constant agitation at room temperature. The sample was kept in the absence of light to avoid degradation of the polyphenols [30]. The extract was then centrifuged in a centrifuge (SL 8R Thermo Fisher Scientific, Waltham, MA, USA) at 1500 rpm for 5 min and the supernatant was filtered. The resulting extract was stored at 4 °C until further analysis. The analyses were performed in triplicate on the MultiSkan Go kit (Thermo Fisher Scientific, USA).

### 2.3. Phenolic Compounds

#### 2.3.1. Total Phenolic Content (TPC)

The determination of the total phenolic content was carried out spectrophotometrically using the Folin–Ciocalteu colorimetric method [31]. The reaction mixture contained 18 µL of the extract, 124.5 µL of deionized water, 37.5 µL of Folin–Ciocalteu reagent (Sigma Aldrich, Schnelldorf, Germany), and 120 µL of 7.1% anhydrous sodium carbonate (Na_2_CO_3_). The extract was left to react for 60 min in the dark at room temperature (24 °C) and the absorbance was read at 760 nm [31]. Gallic acid (Sigma Aldrich, Germany) was used as a standard. The results were expressed as mg gallic acid (GAE)/g of dry plant material.

#### 2.3.2. Total Flavonoid Content (TFC)

The TFC was determined by reaction with aluminum chloride (AlCl_3_) according to the methodology described by Zhishen et al. [32] with slight modifications. The reaction mixture consisted of 120 µL of deionized water, 30 µL of the extract, 9 µL of 5% sodium nitrite (NaNO_2_) (left for 5 min), and 9 µL of 10% aluminum chloride (AlCl_3_) (left for 5 min). Then, 60 µL of 1 M sodium hydroxide (NaOH) (left for 15 min), and finally, 72 µL of deionized water. The reaction mix was left in the dark at room temperature for 30 min and the absorbance was read at 510 nm [32]. The (+)-catechin (Sigma Aldrich, Germany) was used as a standard for the quantification of total flavonoids. Results were expressed as mg catechin (CE)/g of dry plant material.

#### 2.3.3. Total Condensed Tannin Content (TCTC)

The analysis of the anti-nutritional TCTC was based on the reaction with vanillin under acidic conditions according to the methodology described by Butler et al. [33]. A 2 mL aliquot of freshly prepared vanillin solution (1 g/100 mL, Sigma Aldrich, Germany) in 70% sulfuric acid was added to 500 µL of extract. The mixture was incubated at 20 °C for 15 min and its absorbance was read at 500 nm. For the reference curve, (+)-catechin was used as the standard and the results were expressed as mg catechin (EC)/g of dry plant material.

### 2.4. Pigments

#### 2.4.1. Total Monomeric Anthocyanin Content (MAC)

The differential pH method described by Giusti and Wrolstad [34] was used to determine the total monomeric anthocyanin content. This method uses two dilutions of the extract, one obtained from a potassium chloride (KCl) buffer solution at pH 1.0 (0.025 M) and the second from a sodium acetate (CH_3_CO_2_Na-_3_H_2_O) buffer solution at pH 4.5 (0.4 M), which were diluted by the previously determined dilution factor. An absorption spectrum in the range of 460–710 nm was generated to determine the maximum absorbance using a spectrophotometer (Multiskan FC Microplate Photometer, Thermo Fisher Scientific, USA). The monomeric anthocyanin content (MAC) [34] was calculated according to the following equation: AM = A × MW × DF × 1000)/(ε × I). In the equation, the absorbance of sample A corresponds to (Aλ510–Aλ700) pH 1.0 and (Aλ510–Aλ700) pH 4.5; MW = 449.2 is the molecular weight of cyanidin-3-glucoside; ε = 26.900 g/mol is the molar absorbance of cyanidin-3-glucoside; DF is the dilution factor used; and I is the cell length (1 cm). Results were expressed as mg cyanidin-3-glucoside (C3G)/g of dry plant material.

#### 2.4.2. Total Carotenoid Content (TCC)

The carotenoid content was quantified according to the method proposed by Lichtenthaler and Wellburn [35] with some slight modifications. We used 1.0 g of bean seed meal in 6 mL of ice-cooled 99.5% (*v*/*v*) acetone. The sample was vortexed for 10 min and centrifuged at 4500 rpm for another 10 min and the supernatant was used for measurement. Absorbance was measured in quartz cells with a 2 mL volume of extract at 450 nm. The ẞ-carotene was used as a standard for the reference curve and the results were expressed as mg ẞ-carotene/g of dry plant material.

### 2.5. Antioxidant Activity

#### 2.5.1. DPPH Radical Scavenging Activity (DRSA)

The DRSA was used according to the Brand-Williams et al. [36] method with slight modifications. A stock solution (20 mg/L) of DPPH (2,2-Diphenyl-1-picrylhydrazyl) in absolute methanol was prepared. The absorbance of the radicals was adjusted to 0.3 absorbance units with methanol at 4 °C, then 3 µL of the extract and 297 µL of the adjusted DPPH solution were mixed. The reaction mix was kept in the dark for 30 min at room temperature and the absorbance was read at a wavelength of 517 nm [36]. The results were expressed as TEAC (Trolox equivalent antioxidant capacity) values in µmol of Trolox/g of dry plant material, by constructing a reference curve using Trolox as an antioxidant.

#### 2.5.2. ABTS-+ Radical Scavenging Activity (ARSA)

The ARSA was determined according to the method of Re et al. [37] with some modifications. The ABTS-+ (2,2′-azino-bis-(3-ethylbenzothiazoline-6-sulfonic) acid) radical cation was generated by mixing the ABTS stock solution (7 mol/L) with potassium persulfate (2.45 mol/L) and the resulting mixture was allowed to stand in the dark at room temperature for 48 h before use. The ABTS-+ radical solution was diluted in 0.15 mol/L phosphate-buffered saline (PBS) pH 7.4 to obtain an absorbance of 0.7. In the evaluation, 3 µL of the extract and 297 µL of the ABTS-+ radical solution were used. After 30 min of reaction at room temperature and in the dark, the change in absorbance was read with respect to the reagent reference at a wavelength of 734 nm. The results were expressed as TEAC values in µmol of Trolox/g of dry plant material by constructing a reference curve using Trolox as an antioxidant.

#### 2.5.3. Reducing Capacity FRAP (Ferric Reducing Antioxidant Power)

This method is based on evaluating the antioxidant capacity of a sample according to its ability to reduce ferric iron (Fe^3+^) present in a complex with 2,4,6-tri(2-pyridyl)-s-triazine (TPTZ), to the ferrous form (Fe^2+^) [38]. The assay was carried out in a pH 3.6 acetic acid–sodium acetate buffer containing TPTZ and FeCl_3_. Fifteen µL of the extract, 15 µL of buffer, and 270 µL of FRAP solution were mixed and the reaction mix was kept in the dark for 30 min at room temperature. The absorbance was read at a wavelength of 590 nm. FRAP values were expressed as µmol ascorbic acid (AA)/g of dry plant material using ascorbic acid as the standard.

### 2.6. Sugar Content

#### 2.6.1. Total Sugar Content (TSC)

The total sugar content was determined by the phenol-sulfuric colorimetric method according to Dubois et al. [39]. In a 2 mL reaction tube, 210 µL of the extract was mixed, then 200 µL of freshly prepared and shaken phenol 80% was added to an extraction cabinet. Subsequently, 1 mL of 98% (*v*/*v*) concentrated sulfuric acid (Sigma Aldrich, Germany) was added. The reaction mix was vortexed for 1 minute and allowed to cool at room temperature and kept in the dark and read after 15 min at an absorbance of 490 nm. D-glucose (Sigma Aldrich, Germany) was used as a standard. The content was expressed as mg glucose/g dry plant material.

#### 2.6.2. Total Reducing Sugar Content (TRSC)

The content of reducing sugars was determined by the dinitrosalicylic acid (DNS) colorimetric method according to the methodology described by Miller et al. [40]. In a 10 mL reaction tube, 0.5 mL of the methanolic extract was taken, then 0.5 mL of the DNS reagent was added and heated for 15 min in a water bath at a temperature of 99 °C. It was allowed to cool and it was diluted with 5 mL of deionized water. The absorbance was read at a wavelength of 575 nm. D-glucose was used as a standard. The content was expressed as mg glucose/g dry plant material.

### 2.7. Data Analysis

Bean genotypes were grouped using all variables of polyphenols and total and reducing sugar content by cluster analysis. From the bean genotype typologies and using each of the variables, a Principal Component Analysis (PCA) was performed to see the relationship between the variables and their contribution to each of the components. A Monte-Carlo permutation test was used to determine the difference and variance explained between bean genotype types. A linear mixed model (LMM) was then fitted, with typology as the fixed factor and genotype as the random factor to determine the differences in each of the variables between typologies. Within the analysis of variance, using an exploratory analysis of the residuals, the assumptions of normality and homogeneity of variance were evaluated. Differences between typologies were analyzed using the LSD Fisher post hoc test with a significance of α = 0.05. A correlation analysis was also performed using Pearson’s test to determine the relationship between the different variables, the visualization of which was performed using a grid where the thickness of the line was considered the magnitude of the positive (blue) and negative (red) correlation. In order to determine the range of nutritional quality of the 112 bean genotypes evaluated, an indicator was proposed that had the capacity to express nutritional quality (NQI) [41]. The NQI is obtained from the transformation of each of the variables ranging from 0 (minimum) to 1 (maximum) under the criteria of the following: (i) more is better, suitable for standardizing the scores of the properties of the bean genotypes which were associated with values close to one (1); (ii) less is worse, those properties whose values were close to zero (0), an approach that has been used in other studies. This NQI indicator has the capacity to explain what is good or not due to the transformation of the variables, taking each one of the variables to the range (0–1) of values where a higher value is given to the variables that contribute positively to nutrition and the anti-nutritional ones would have the lowest value. Clustering and PCA were performed using the fviz_dend and fviz_pca_ind functions, respectively, from the “factoextra” package; the LMM was performed using the lme function in the “nlme” package; and the graphical outputs were performed in the “ade4”, “ggplot2”, “factoextra”, and “corrplot” packages in R language software, version 4.2.0 [42], using the RStudio interface [42].

## 3. Results

**Figure 1:**
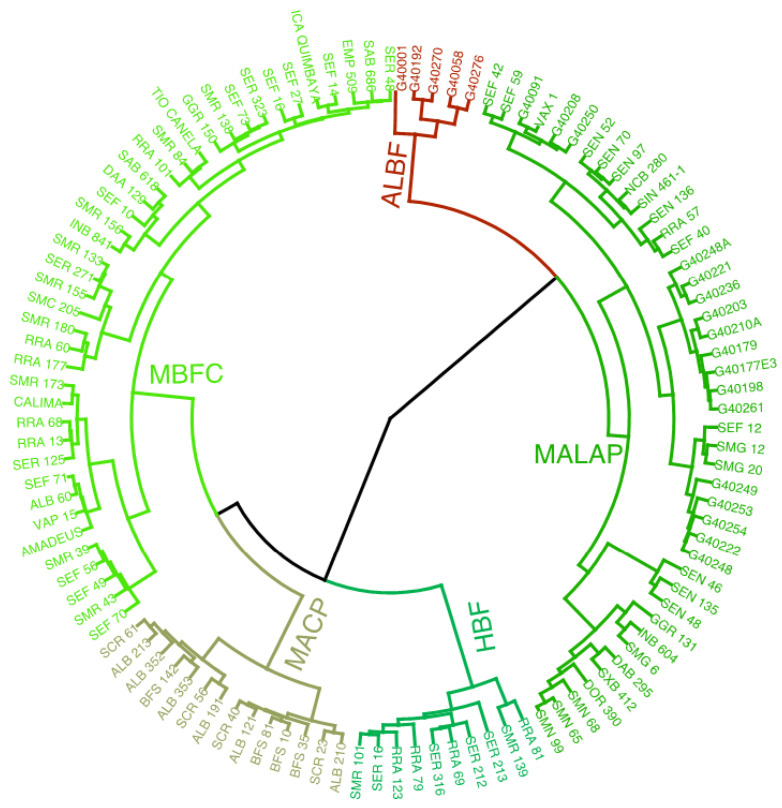
Typology of common bean genotypes grouped by dry seed bioactive component content and antioxidant activity (Ward method, Euclidean distance). HBF: highly bioactive and functional; MBFC: moderately bioactive and functional content; MACP: moderate antioxidant content with pigment influence; MALAP: moderately antinutritional with limited antioxidant potential; and ALBF: antinutritional, low bioactive, and functional.

According to the cluster analysis of different variables determined from the 112 bean genotypes, 5 statistically different groups were identified (Figure 1). According to the content of bioactive components and antioxidant activity, the bean genotypes were divided into five typologies: (1) highly bioactive and functional (HBF); (2) moderately bioactive and functional content (MBFC); (3) moderate antioxidant content with pigment influence (MACP); (4) moderately antinutritional with limited antioxidant potential (MALAP); and (5) antinutritional, low bioactive, and functional (ALBF).

**Figure 2:**
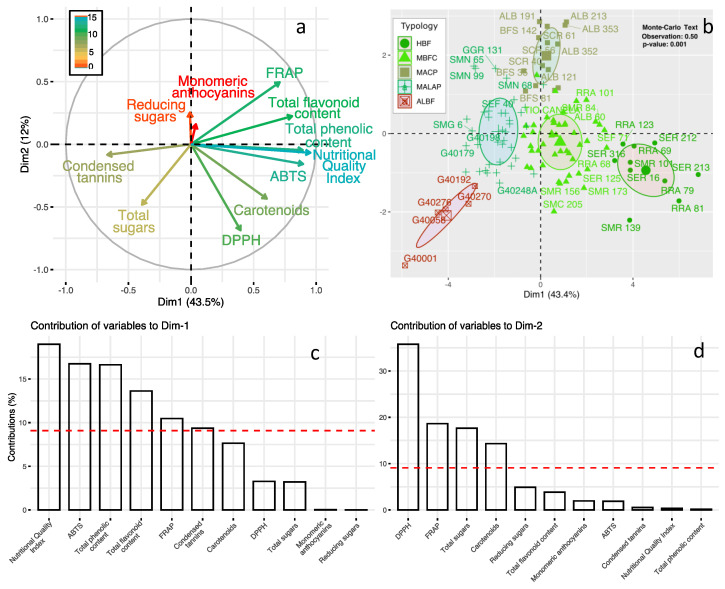
PCA projection of the variables related to seed bioactive components and antioxidant activity grouped by the type of common bean genotypes. (**a**) Correlation circle between the variables of bioactive component content and antioxidant activity; (**b**) typologies of common bean genotypes; (**c**,**d**) contribution of physiological variables to the formation of the PC1/PC2 principal components of the PCA under different types of common bean genotypes; variables above the red line are significant contributors. Gradient from blue to red means a contribution from higher to lower. HBF: highly bioactive and functional; MBFC: moderately bioactive and functional content; MACP: moderate antioxidant content with pigment influence; MALAP: moderately antinutritional with limited antioxidant potential; and ALBF: antinutritional, low bioactive, and functional.

The results of the Principal Component Analysis (PCA, Figure 2a) axis 1 (43.5% of the variance can be explained) separates bean genotypes related to the high seed NQI, ABTS, total phenolic content, total flavonoid content, and FRAP with those that contain high tannin content. Axis 2 (12% of explained variance, Figure 2a) opposes genotypes with characteristics related to DPPH, carotenoids, and total sugars with those having high FRAP content. According to the Monte-Carlo test (Figure 2b), the separation of bean genotype typologies according to physiological ones was significant and it explained 50% of the total variance according to the Monte-Carlo test (Figure 2b). The variables with the highest contribution in axes 1 and 2 are shown in Figure 2c,d, respectively.

Below, we describe the representative characteristics of each type of bean genotype. The main differences are due to the total phenolic content, ABTS, as well as NQI values (Table 1). The monomeric anthocyanin was the only one that did not show a statistical difference between the typologies (Table 1).

Highly bioactive and functional (HBF; *n* = 10; 8.9% of the total genotypes evaluated). This typology is characterized by having higher contents of total phenolic content, total flavonoid content, DPPH, FRAP, ABTS, carotenoids, and a lower content of condensed tannins, compared to the other typologies, thus allowing it to have on an average the higher NQI with values around 0.74. Genotypes such as SER 212, SER 213, and RRA 81 were found to be outstanding in this typology, showing NQI values above 0.8. Interestingly, the materials with greater NQI values came from Mesoamerican lines resulting from the crosses between *P. vulgaris* × *P. acutifolius* with red grain, as is the case of SER, and the Andean RRA came from the cross between *P. vulgaris* × *P. coccineus,* again with red grain.

Moderately bioactive and functional content (MBFC; *n* = 40; 35.7%). This big typology group consisted of genotypes that had values above the overall average in variables such as total phenolic content, total flavonoid content, DPPH, FRAP, ABTS, and carotenoids, which influenced a low average of NQI values. SEF 10 genotype (red-seeded Mesoamerican), whose NQI value was 0.59, resulted from the cross of *P. vulgaris* × *P. coccineus* × *P. acutifolius.* This line was outstanding in this typology group, with a higher NQI value.

Moderate antioxidant content with pigment influence (MACP; *n* = 14; 12.5%). This typology group consists of lines whose values—for most of the variables evaluated—were very close to the overall mean, with the exceptions of DPPH and FRAP. These two traits presented the lowest and highest values, respectively, compared to other typologies (*p* < 0.05). This group contains mostly lines such as ALB, BFS, and SCR, whose NQI values were low compared to the other typologies (*p* < 0.05). From this typology group, we highlight materials such as ALB 353, ALB 352, and ALB 213, whose NQI values were higher than 0.45. These genotypes with red grains and of Mesoamerican origin were derived from the crosses of *P. vulgaris* × *P. acutifolius*.

Moderately antinutritional with limited antioxidant potential (MALAP; *n* = 43; 38.3%). The bean genotypes that are part of this biggest typology group had low NQI values compared to the other typologies (*p* < 0.05). The values of the nutritional variables were below the general average. However, the condensed tannin content was 41% higher than the general average. Of this group, we highlight materials such as SEN 48, SEN 135, and G40249 (*P. acutifolius*), whose NQI values were higher than 0.36.

Antinutritional, low bioactive, and functional (ALBF; *n* = 5; 4.4%). This typology is characterized by genotypes with high contents of total sugars, condensed tannins, and lower values of reducing sugars, total phenolic content, total flavonoid content, FRAP, and ABTS. Taken together, this resulted in a very low NQI value, with the average being only 0.11. For example, in the case of total sugar content, this typology group showed values three times higher compared to the general average. A similar situation occurred in the condensed tannin content, for which the value was 144% higher compared to the general average, with G40001 and G40058 (*P. acutifolius*) having very low NQI values (0.08).

**Figure 3:**
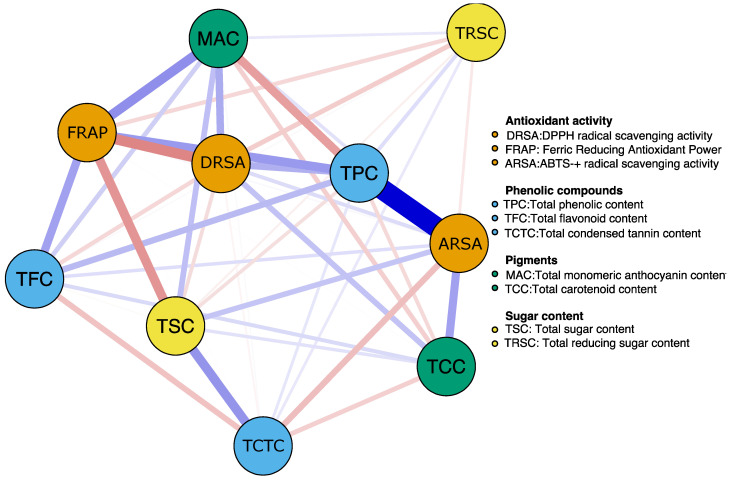
Correlation network between the different variables of antioxidant activity, phenolic compounds, pigments, and sugar content evaluated in different bean materials. Positive and negative correlations are represented with blue and red colors, respectively. The magnitude of the correlation level corresponds to the thickness of the line. A thicker line indicates a stronger correlation.

According to the results obtained from the Pearson correlation analysis (Figure 3), it was found that the TPC presented a high positive correlation with the ARSA, TFC, and FRAP (r > 0.75; *p* < 0.05). Likewise, the TFC presented a moderate positive correlation with the ARSA and FRAP (r > 0.60; *p* < 0.05). However, the ARSA was negatively correlated with the TCTC (r = −0.51; *p* < 0.05), and the latter was also negatively correlated with the TFC and TPC (r = −0.44; *p* < 0.05).

**Figure 4:**
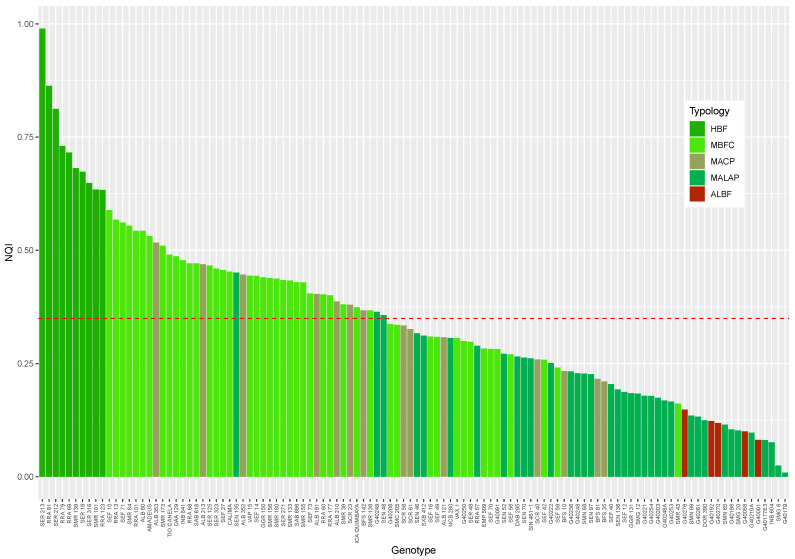
Nutritional quality index (NQI) of common bean genotypes grown in the western Amazon region under high temperatures and acid soil stress conditions. HBF: highly bioactive and functional; MBFC: moderately bioactive and functional content; MACP: moderate antioxidant content with pigment influence; MALAP: moderately antinutritional with limited antioxidant potential; and ALBF: antinutritional, low bioactive, and functional. The red horizontal line shows the overall population mean of the NQI (0.34).

When analyzing the chemical composition in terms of the antioxidant activity, phenolic compounds, pigments, sugar content, and using the NQI values, we found differences among genotypes (Figure 4). For example, many genotypes were below the overall mean (NQI = 0.34; mainly from typology groups of MALAP and ALBF). Thus, the higher temperature and acid soil conditions in the western Amazon make them susceptible to heat, resulting in reduced grain quality. However, lines such as RRA (RRA 69, RRA 79, RRA 81) SER (SER 16, SER 212, SER 213, SER 316), and SMR (SMR 101, SMR 139) showed NQI values above 0.73 due to their ability to adapt to acid soil conditions and high temperature stress.

## 4. Discussion

Using the NQI as a method of quantitative and qualitative analysis of bean seeds, we identified some bean genotypes with exceptionally high nutritional quality under high temperatures and acid soil stress conditions of the western Amazon region. High NQI values mean that some lines had high contents of total phenols, total flavonoids, DPPH, FRAP, ABTS, and carotenoids. These nutritional characteristics present beans as a significant source of natural antioxidants which correlate directly with the content of phenolic compounds [43].

The content of nutritional compounds has been reported to depend on the bean species [13,44] as well as on the capacity for mineral absorption, assimilate translocation, and capacity of storage of bioactive compounds in the cotyledons and bean seed coat [3]. These differences between species have been documented by different authors [45,46], who mentioned that the consumption of species of the genus *Phaseolus* has important nutritional and functional contributions to human health. This is specifically related to the contents of catechins (flavonoids, mg CE g^−1^), gallic acid (phenols, mg GAE g^−1^), and cyanidin (anthocyanins, mg C3G g^−1^) [3]. Higher nutritional values were found in nine common bean lines including RRA (RRA 69, RRA 79, RRA 81), SER (SER 16, SER 212, SER 213, SER 316), and SMR (SMR 101, SMR 139). These bean lines are all with red seed coat (testa), whose antioxidant activity in the HBF typology group was generally higher compared to the other four typology groups.

In this sense, the color of the seed coat can be a very important variable when selecting materials in genetic improvement programs for improving nutritional quality [13,47,48]. However, this relationship needs further analysis to improve current understanding, especially under different environments to dissect the genotype by environment (G × E) interactions on improving the nutritional quality of bean seeds. This is because seed quality also depends on the production of secondary metabolites that are induced by changes in environmental conditions. A large proportion of phenols, flavonoids, and condensed tannins are stored in the canopy biomass, which correlates with antioxidant activity, and these compounds influence the change in color of the seed coat [49,50,51]. Therefore, a higher content of these compounds and antioxidant activity were mainly presented in bean seeds with red testa in lines derived from crosses between *P. vulgaris* × *P. acutifolius* (SER) and also in lines derived from crosses between *P. vulgaris* × *P. coccineus* (RRA) compared to those genotypes with white testa, such as *Phaseolus acutifolius* (G40001 and G40058). This was well documented by other published studies [20,52,53,54]. Taking the published reports and the results from this study together, we suggest that the antioxidant potential depends on the phenols, flavonoids, and condensed tannins contained both in the seed and in the seed coat [20].

Our results showed that five genotypes have high phenolic contents and these include RRA 79 (19.65 mg g^−1^, red), RRA 81 (16.03 mg g^−1^, red), SER 213 (19.03 mg g^−1^, red), SER 212 (17.58 mg g^−1^, red), SEF 71 (13.87 mg g^−1^, red), and ALB 60 (12.49 mg g^−1^, red), indicating the importance of beans as a source of these secondary metabolites (phenols, flavonoids, anthocyanins, and tannins, among others) [55,56,57]. These compounds possess functional properties due to the ability to neutralize reactive oxygen species, thus counteracting the initiation and propagation of oxidative processes, preventing the incidence of diseases caused by this imbalance [20,21,58]. Added to what was said above, the high contents of secondary metabolites were positively correlated with compounds related to antioxidant activity measured through ABTS radical and iron-reducing power (FRAP) techniques.

Antioxidant compounds that influence bean testa color include anthocyanin content, which confers a variety of colors ranging from red to black to pink [20]. Variations in anthocyanin contents have been found, specifically in the seed coat of species such as *P. vulgaris* and *P. coccineus* [59,60]. However, in our study, no significant variation was found among the evaluated genotypes, which is probably due to a homogeneous distribution of compounds in both the seed coat and cotyledon [3]. Regarding carotenoids, bean genotypes that reached maximum values of this parameter were SMR 139 (11.74 mg g^−1^), RRA 81 (10.28 mg g^−1^), SMR 133 (6.20 mg g^−1^), SER 271 (5.45 mg g^−1^), and RRA 69 (4.77 mg g^−1^). The importance of having high contents of carotenoids or 𝛽-carotene in beans lies in the possibility to metabolize and transform them into vitamin A. They are also essential in the formation of rhodopsin, which plays an important role in visual adaptation to light changes, cell replication, and tissue homeostasis [61,62,63]. On the other hand, high carotenoid contents present a strong statistical correlation with antioxidant behavior, which is measured through the change in DPPH radicals. This can be explained by the hydrophobic character of the molecular structure and the capacity of 𝛽-carotene to eliminate reactive oxygen species [64].

Regarding the total sugars content, higher values were found in four *P. acutifolius* materials (G40001 (702.1 mg g^−1^), G40058 (559.4 mg g^−1^), G40276 (524.7 mg g^−1^), G40192 (330.5 mg g^−1^)), followed by *P. vulgaris* (SAB 618 (346.6 mg g^−1^)), and in two lines from the crosses of *P. vulgaris* × *P. acutifolius* (INB 841 (320.1 mg g^−1^)) and of *P. vulgaris* × *P. coccineus* × *P. acutifolius* (SEF 10 (255.99 mg g^−1^)). The reducing sugars in lines from crosses of *P. vulgaris* × *P. coccineus* × *P. acutifolius* showed higher values (SEF 12 (0.46 mg g^−1^), SEF 10 (0.43 mg g^−1^)), followed by the materials from crosses of *P. vulgaris* × *P. coccineus* (ALB 353 (0.44 mg g^−1^), SER 212 (0.38 mg g^−1^), RRA 69 (0.37 mg g^−1^)). These nutritional parameters indicate the contribution of beans as a source of energy in the diet and, therefore, beans are recognized as a fundamental diet for improving human nutrition [65,66]. Furthermore, the sugar content can influence the organoleptic properties of flavor by activating chemoreceptors on the human tongue or by modifying the flavor of other organic components. The sugar content can even be used as a parameter to differentiate between species and cultivars. For example, *P. vulgaris* is considered as sweet [67,68], since sugar levels in common bean depend on both the genotype and the environmental conditions where it is grown [69,70,71]. *P acutifolius* genotypes tested in our study presented higher values of total glucose. Glucose represents a significant source of metabolic energy for seedling development, in the formation of nutrients and bioactive compounds contained in the seed [72,73], and these compounds are also mainly bound to sugar molecules (glycosylated residues) and proteins [74].

Finally, in terms of human health benefits, bean consumption can prevent different diseases [75,76,77], in addition to the contribution of bioactive components and antioxidant activity found in different genotypes evaluated in this study. For example, in a typical Mediterranean diet based on foods such as olive oil, fruits, vegetables, cereals, legumes, wine, among others, a daily intake per person of 118.6 mg of flavonoids, spiced in flavanones 32% (38.5 mg), catechins (32.7 mg), flavonols 22% (26.4 mg), anthocyanidins 9% (11 mg), flavones 8% (8.7 mg), isoflavones 1% (1.3 mg) [78], and even an ABTS antioxidant potential of around 3500–5300 Trolox equivalents is estimated [79]. While in our study, the genotypes comprising the HBF and MBFC typology groups presented mean values of total flavonoids of 15.15 and 13.23 mg EC g^−1^, respectively, and also an antioxidant potential of 1792 and 974 µmol Trolox g^−1^, respectively. We found four genotypes including SER 213, SMR 101, RRA 81, and RRA 123 of the HBF typology group and six genotypes including ALB 60, AMADEUS, GGR 150, SEF 71, SMR 84, and TIO CANELA of the MBFC typology group with high contents of total flavonoids. We also found seven bean genotypes with high antioxidant activity (ABTS) including SER 213, RRA 79, RRA 81, RRA 69, SER 212, SER 16, and RRA 123 of the HBF typology group, and four genotypes including RRA 13, RRA 68, and SMR 173 of the MBFC typology group. This indicates that 100 g of seeds can provide about 10 times more flavonoid content and supply with great amplitude the daily antioxidant intake compared to the traditional Mediterranean diet, contributing to the prevention of diseases associated with oxidative stress and possibly even exerting antimutagenic/antigenotoxic properties [78].

## 5. Conclusions

In this study, we analyzed the content of bioactive compounds and antioxidant activity in seeds of 118 bean genotypes grown under high temperatures and acid soil stress conditions of western Amazonia. Germplasm accessions of *P. acutifolius* showed lower values of nutritional quality in seeds. Based on the results obtained on bioactive compounds and functional capacity, we classified the bean genotypes into five typology groups including (1) highly bioactive and functional (HBF); (2) moderately bioactive and functional (MBF); (3) moderate antioxidant content with pigment influence (MACP); (4) moderately antinutritional with limited antioxidant potential (MALAP); and (5) antinutritional, low bioactive and functional (ALBF). The typology group of HBF exhibited greater nutritional quality based on nutritional quality index (NQI) values. Three interspecific bred lines (SER 212, SER 213, and RRA 81) belonging to the HBF typology group showed greater NQI values because of the greater level of bioactive compounds and antioxidant capacity. These three bred lines also showed higher contents of total phenolic compounds; total flavonoids; 2,2-diphenyl-1-picrylhydrazyl radical (DPPH); ferric reducing antioxidant power (FRAP); 2,2-azino-bis(3-ethylbenzothiazoline-6-sulfonic acid)diammonium salt (ABTS); carotenoids; and lower content of condensed tannins. For developing nutritionally superior and stress-resilient beans, these three bred lines may be useful as parents in the ongoing bean breeding programs. These improved bred lines may also be useful for developing novel bean-based food formulations. Further research is needed to define the role of testa color in improving the nutritional quality of seeds of common bean genotypes grown under different climatic conditions.

## Figures and Tables

**Table 1:** Bioactive compounds and antioxidant activity of different typology groups of bean genotypes. HBF: highly bioactive and functional; MBFC: moderately bioactive and functional content; MACP: moderate antioxidant content with pigment influence; MALAP: moderately antinutritional with limited antioxidant potential; and ALBF: antinutritional, low bioactive, and functional.

Variable	HBF	MBFC	MACP	MALAP	ALBF	Overall Average	*p*-Value
Total sugars	126.05 ± 13.81 ^b^	147.61 ± 10.49 ^b^	137.29 ± 9.23 ^b^	146.31 ± 10.08 ^b^	505.13 ± 63.99 ^a^	159.93 ± 9.43	<0.0001
Reducing sugars	0.3 ± 0.02 ^a^	0.28 ± 0.01 ^a^	0.3 ± 0.02 ^a^	0.32 ± 0.01 ^a^	0.24 ± 0.02 ^b^	0.3 ± 0.01	0.0002
Total phenolic content	15.77 ± 0.86 ^a^	10.05 ± 0.25 ^d^	9.35 ± 0.3 ^c^	7.06 ± 0.22 ^b^	5.14 ± 0.53 ^e^	9.26 ± 0.28	<0.0001
Total flavonoid content	15.15 ± 0.53 ^a^	12.85 ± 0.34 ^c^	12.61 ± 0.43 ^b^	8.57 ± 0.31 ^b^	6.83 ± 0.25 ^d^	11.34 ± 0.3	<0.0001
DPPH	3068.68 ± 104.56 ^a^	2501.95 ± 37.61 ^c^	1126.2 ± 32.84 ^d^	2250.97 ± 80.3 ^b^	2167.89 ± 78.07 ^c^	2282.76 ± 56.75	<0.0001
FRAP	7.19 ± 0.35 ^a^	5.15 ± 0.17 ^c^	6.93 ± 0.37 ^a^	3.74 ± 0.24 ^b^	1.85 ± 0.42 ^d^	4.94 ± 0.18	<0.0001
ABTS	1791.85 ± 133.61 ^a^	945.56 ± 38.26 ^c^	827.89 ± 36.2 ^c^	546.25 ± 29.49 ^b^	329.23 ± 38.2 ^e^	846.98 ± 40.24	<0.0001
Carotenoids	5.49 ± 0.96 ^a^	2.85 ± 0.22 ^c^	2.04 ± 0.2 ^c^	1.53 ± 0.17 ^b^	1.5 ± 0.23 ^c^	2.49 ± 0.17	<0.0001
Condensed tannins	1.1 ± 0.15 ^c^	1.48 ± 0.08 ^b^	1.54 ± 0.1 ^c^	3.49 ± 0.27 ^c^	5.59 ± 1.61 ^a^	2.29 ± 0.16	<0.0001
Monomeric anthocyanins	0.01 ± 0	8.8 ± 0.56	10.34 ± 0.79	0.01 ± 0	10.58 ± 1.02	8.82 ± 0.28	
NQI	0.74 ± 0.04 ^a^	0.41 ± 0.01 ^d^	0.35 ± 0.03 ^c^	0.2 ± 0.02 ^b^	0.11 ± 0.01 ^c^	0.35 ± 0.02	<0.0001

Mean ± Standard error. ^a,b,c,d,e^: averages with a letter in common between columns are not significantly different at 5% probability. DPPH: 2,2-Diphenyl-1-picrylhydrazyl; FRAP: ferric-reducing antioxidant power assay; ABTS: (2,2′-azino-bis-(3-ethylbenzothiazoline-6-sulfonic) acid); NQI: nutritional quality index.

## Data Availability

The data presented in this study are available on request from the corresponding author.

## Supplementary Materials

The following supporting information can be downloaded at https://www.mdpi.com/article/10.3390/foods12152849/s1, Supplement Table S1. List of common bean genotypes used in the study.
