# Supplementary material for: Bioactive Compounds and Antioxidant Activity in Seeds of Bred Lines of Common Bean Developed from Interspecific Crosses
Source: Foods. 2023 Jul 27;12(15):2849. doi: 10.3390/foods12152849 (PMC10417468; doi:10.3390/foods12152849)
Supplement: Supplementary file 1 [file foods-12-02849-s001.zip › foods-2498322-supplementary.pdf]

Supplement 1. List of common bean genotypes used in the study

Genotype	Commercial grain color	Genepool	Growth habit	Classification	Cross within or between species
ALB 121	Brown	Mesoamerican	2B	Inbred line	Pv x Pc
ALB 191	Black	Mesoamerican	2B	Inbred line	Pv x Pc
ALB 210	Red	Mesoamerican	2A	Inbred line	Pv x Pc
ALB 213	Red	Mesoamerican	2B	Inbred line	Pv x Pc
ALB 352	Red	Mesoamerican	2B	Inbred line	Pv x Pc
ALB 353	Red	Mesoamerican	2A	Inbred line	Pv x Pc
ALB 60	Red	Mesoamerican	2B	Inbred line	Pv x Pc
Amadeus	Red	Mesoamerican	2A	Inbred line	Pv
BFS 10	Red	Mesoamerican	2A	Inbred line	Pv
BFS 142	Red	Mesoamerican	2B	Inbred line	Pv x Pc
BFS 35	Red	Mesoamerican	2B	Inbred line	Pv
BFS 81	Red	Mesoamerican	2B	Inbred line	Pv x Pc
Calima	Red mottled	Andean	1	Inbred line	Pv
DAA 129	Red pink	Andean	2B	Inbred line	Pv
DAB 295	Red mottled	Andean	1	Inbred line	Pv
DOR 390	Black	Mesoamerican	2B	Inbred line	Pv
EMP 509	Cream striped	Mesoamerican	2A	Inbred line	Pv
G40001	White	Mesoamerican	4A	Inbred line	Pa
G40058	White	Mesoamerican	4A	Inbred line	Pa
G40091	Brown small	Mesoamerican	4A	Inbred line	Pa
G40177E3	Brown small	Mesoamerican	4A	Inbred line	Pa
G40179	Brown small	Mesoamerican	4A	Inbred line	Pa
G40192	Brown small	Mesoamerican	4A	Inbred line	Pa
G40198	Brown small	Mesoamerican	4A	Inbred line	Pa
G40203	Brown small	Mesoamerican	4A	Inbred line	Pa
G40208	Brown small	Mesoamerican	4A	Inbred line	Pa
G40210A	Brown small	Mesoamerican	4A	Inbred line	Pa
G40221	Brown small	Mesoamerican	4A	Inbred line	Pa
G40222	Brown small	Mesoamerican	4A	Inbred line	Pa

Genotype	Commercial grain color	Genepool	Growth habit	Classification	Cross within or between species
G40236	Brown small	Mesoamerican	4A	Inbred line	Pa
G40248	Brown small	Mesoamerican	4A	Inbred line	Pa
G40248A	Brown small	Mesoamerican	4A	Inbred line	Pa
G40249	Brown small	Mesoamerican	4A	Inbred line	Pa
G40250	Black small	Mesoamerican	4A	Inbred line	Pa
G40253	Brown small	Mesoamerican	4A	Inbred line	Pa
G40254	Brown small	Mesoamerican	4A	Inbred line	Pa
G40261	Brown small	Mesoamerican	4A	Inbred line	Pa
G40270	Orange mottled	Mesoamerican	4A	Inbred line	Pa
G40276	White	Mesoamerican	4A	Inbred line	Pa
GGR 131	Red	Mesoamerican	2A	Inbred line	Pv
GGR 150	Cream red mottled	Mesoamerican	2B	Inbred line	Pv x Pc x Pa x Pd
Ica Quimbaya	Red	Andean	1	Commercial variety	Pv
INB 604	Black	Mesoamerican	2A	Inbred line	Pv x Pa
INB 841	Brown speckled	Mesoamerican	2A	Inbred line	Pv x Pa
NCB 280	Black	Mesoamerican	2B	Inbred line	Pv
RRA 13	Red	Andean	2B	Inbred line	Pv x Pc
RRA 57	Red mottled	Andean	1	Inbred line	Pv x Pc
RRA 60	Red mottled	Andean	1, 2A	Inbred line	Pv x Pc
RRA 68	Red	Andean	2A	Inbred line	Pv x Pc
RRA 69	Pink	Andean	2A	Inbred line	Pv x Pc
RRA 79	Red	Andean	2A	Inbred line	Pv x Pc
RRA 81	Red	Andean	2A	Inbred line	Pv x Pc
RRA 101	Red	Andean	2B	Inbred line	Pv x Pc
RRA 123	Pink	Andean	2B	Inbred line	Pv x Pc
RRA 177	Red	Andean	2A	Inbred line	Pv x Pc
SAB 618	Red mottled	Andean	1	Inbred line	Pv
SAB 686	Cream mottled	Andean	1	Inbred line	Pv
SCR 23	Red	Mesoamerican	2B	Inbred line	Pv
SCR 40	Red	Mesoamerican	2B	Inbred line	Pv

Genotype	Commercial grain color	Genepool	Growth habit	Classification	Cross within or between species
SCR 56	Red	Mesoamerican	2B	Inbred line	Pv
SCR 61	Red	Mesoamerican	2B	Inbred line	Pv
SEF 10	Red	Mesoamerican	2A	Inbred line	Pv x Pc x Pa
SEF 12	Red	Mesoamerican	2A	Inbred line	Pv x Pc x Pa
SEF 14	Red	Mesoamerican	2A	Inbred line	Pv x Pc x Pa
SEF 16	Red	Mesoamerican	2A	Inbred line	Pv x Pc x Pa
SEF 27	Red	Mesoamerican	2B	Inbred line	Pv x Pc x Pa
SEF 40	Red	Mesoamerican	2A	Inbred line	Pv x Pc x Pa
SEF 42	Red	Mesoamerican	2A	Inbred line	Pv x Pc x Pa
SEF 49	Red	Mesoamerican	2A	Inbred line	Pv x Pc x Pa
SEF 56	Red	Mesoamerican	2B	Inbred line	Pv x Pc x Pa
SEF 59	Red	Mesoamerican	2A	Inbred line	Pv x Pc x Pa
SEF 70	Red	Mesoamerican	2B	Inbred line	Pv x Pc x Pa
SEF 71	Red	Mesoamerican	2A	Inbred line	Pv x Pc x Pa
SEF 73	Red	Mesoamerican	2B	Inbred line	Pv x Pc x Pa
SEN 46	Black	Mesoamerican	2B	Inbred line	Pv
SEN 48	Black	Mesoamerican	2B	Inbred line	Pv
SEN 52	Black	Mesoamerican	2A	Inbred line	Pv
SEN 70	Black	Mesoamerican	2B	Inbred line	Pv
SEN 97	Black	Mesoamerican	2B	Inbred line	Pv
SEN 135	Black	Mesoamerican	2A	Inbred line	Pv
SEN 136	Black	Mesoamerican	2A	Inbred line	Pv
SER 16	Red	Mesoamerican	2A	Inbred line	Pv
SER 48	Red	Mesoamerican	2B	Inbred line	Pv
SER 125	Red	Mesoamerican	2B	Inbred line	Pv
SER 212	Red	Mesoamerican	2A	Inbred line	Pv x Pa
SER 213	Red	Mesoamerican	2A	Inbred line	Pv x Pa
SER 271	Red	Mesoamerican	2A	Inbred line	Pv
SER 316	Red	Mesoamerican	2A	Inbred line	Pv
SER 323	Red	Mesoamerican	2A	Inbred line	Pv

Genotype	Commercial grain color	Genepool	Growth habit	Classification	Cross within or between species
SIN 461-1	Brown	Mesoamerican	2B	Inbred line	Pv x Pa
SMC 205	Red mottled	Mesoamerican	2B	Inbred line	Pv x Pc x Pa x Pd
SMG 12	Pink	Mesoamerican	2A	Inbred line	Pv x Pc x Pa x Pd
SMG 6	Pink	Mesoamerican	2A	Inbred line	Pv x Pc x Pa x Pd
SMG 20	Red Pink mottled	Mesoamerican	2B	Inbred line	Pv x Pc x Pa x Pd
SMN 65	Black	Mesoamerican	2B	Inbred line	Pv
SMN 68	Black	Mesoamerican	2B	Inbred line	Pv
SMN 99	Black	Mesoamerican	2B	Inbred line	Pv
SMR 39	Red	Mesoamerican	2A	Inbred line	Pv
SMR 43	Red	Mesoamerican	2B	Inbred line	Pv
SMR 84	Red	Mesoamerican	2B	Inbred line	Pv x Pd
SMR 101	Red	Mesoamerican	2B	Inbred line	Pv
SMR 133	Red	Mesoamerican	2A	Inbred line	Pv
SMR 139	Red	Mesoamerican	2B	Inbred line	Pv
SMR 155	Red	Mesoamerican	2A	Inbred line	Pv
SMR 156	Red	Mesoamerican	2B	Inbred line	Pv
SMR 173	Red	Mesoamerican	2B	Inbred line	Pv
SMR 180	Red	Mesoamerican	2B	Inbred line	Pv
SMR 138	Red	Mesoamerican	2B	Inbred line	Pv
SXB 412	Cream	Mesoamerican	2B	Inbred line	Pv
Tio Canela	Red	Mesoamerican	2A	Inbred line	Pv
VAP 15	Red	Mesoamerican	2B	Inbred line	Pv x Pa x Pparv
VAX 1	Cream striped	Mesoamerican	3B	Inbred line	Pv

Pv: *Phaseolus vulgaris*, Pc: *Phaseolus coccineus*, Pa: *Phaseolus acutifolius*, Pd: *Phaseolus dumosus*. Growth habit type I is an erect determinate bush, growth habit type IIA is an erect, indeterminate bush with short guide, growth habit type IIB is a semierect, indeterminate bush.
